# Biochemical and functional characterization of *Brucella abortus* cyclophilins: So similar, yet so different

**DOI:** 10.3389/fmicb.2022.1046640

**Published:** 2022-10-31

**Authors:** Emanuel J. Muruaga, Gabriel Briones, Mara S. Roset

**Affiliations:** ^1^Instituto de Investigaciones Biotecnológicas, Universidad Nacional de San Martín (UNSAM)-Consejo Nacional de Investigaciones Científicas y Técnicas (CONICET), Buenos Aires, Argentina; ^2^Escuela de Bio y Nanotecnologías (EByN), Universidad Nacional de San Martín, Buenos Aires, Argentina

**Keywords:** brucellosis, *Brucella abortus*, Cyclophilins, virulence, PPIase activity, dimeric CypB, Brucella-host interaction, stress adaptation

## Abstract

*Brucella* spp. are the etiological agent of animal and human brucellosis. We have reported previously that cyclophilins of *Brucella* (CypA and CypB) are upregulated within the intraphagosomal replicative niche and required for stress adaptation and host intracellular survival and virulence. Here, we characterize *B. abortus* cyclophilins, CypA, and CypB from a biochemical standpoint by studying their PPIase activity, chaperone activity, and oligomer formation. Even though CypA and CypB are very similar in sequence and share identical chaperone and PPIase activities, we were able to identify outstanding differential features between them. A series of differential peptide loops were predicted when comparing CypA and CypB, differences that might explain why specific antibodies (anti-CypA or anti-CypB) were able to discriminate between both cyclophilins without cross-reactivity. In addition, we identified the presence of critical amino acids in CypB, such as the Trp^134^ which is responsible for the cyclosporin A inhibition, and the Cys^128^ that leads to CypB homodimer formation by establishing a disulfide bond. Here, we demonstrated that CypB dimer formation was fully required for stress adaptation, survival within HeLa cells, and mouse infection in *B. abortus*. The presence of Trp^134^ and the Cys^128^ in CypB, which are not present in CypA, suggested that two different kinds of cyclophilins have evolved in *Brucella*, one with eukaryotic features (CypB), another (CypA) with similar features to Gram-negative cyclophilins.

## Introduction

Cyclophilins are enzymes that belong to the superfamily of peptidyl-prolyl cis/trans isomerases (PPIases: EC 5.2.1.8). These enzymes act as biological catalysts speeding up the rate-limiting cis/trans or trans/cis conformational changes at Xaa-Pro bonds during protein folding in both eukaryotes and prokaryotes. The spontaneous isomerization of the peptidyl proline bonds is a slow reaction and consequently requires the assistance of PPIases that accelerates this step during protein folding. In addition to the cyclophilins, the superfamily of PPIases, also includes the FK506-binding proteins (FKBPs) and the parvulins, a classification that is based on their structure and specific inhibitor compound. Thus, while cyclophilins are inhibited by the immunosuppressive cyclosporin A (CsA), the FKBPs and parvulins are inhibited by the compounds FK506, and rapamycin, respectively (Galat, 2003; Fanghanel and Fischer, 2004).

Cyclophilins are either small single-domain proteins or large multi-domain ones (Pemberton, 2006; Krucken et al., 2009). In the case of the multi-domain cyclophilins, it has been described that additionally to the cyclophilin domain there are also protein domains that act as chaperones or promote the oligomerization state of the protein. Interestingly, even though single-domain cyclophilins are devoid of a canonical chaperone protein domain they can still present certain chaperone activity, which in some cases it has been shown to be independent of the PPIase catalytic activity (Dimou et al., 2011; Zhang et al., 2013; Pandey et al., 2016). Moreover, it has been described that some of the single-domain cyclophilins are still able to oligomerize (Zhang et al., 2011; Jakob et al., 2016).

Of relevance, the over-expression of many cyclophilin-encoding genes is triggered in response to a variety of stressors, suggesting a possible function of cyclophilins (Cyps) in stress adaptation. In agreement with this, microbial Cyps have been described to improve microbial survival under stress conditions and to be upregulated upon host-cell internalization, suggesting a possible function of these proteins in microbial-host interaction (Dimou et al., 2017). Interestingly, the critical role of PPIases in stress tolerance and pathogenesis of bacteria has been demonstrated in *Yersinia pseudotuberculosis* (Obi et al., 2011), *Streptococcus pneumoniae* (Hermans et al., 2006), *Enterococcus faecalis* (Reffuveille et al., 2012), *Streptococcus gordoni* (Cho et al., 2013), *Mycobacterium tuberculosis* (Pandey et al., 2017), *Staphylococcus aureus* (Wiemels et al., 2017; Keogh et al., 2018), *Legionella pneumophila* (Rasch et al., 2019), *Burkholderia pseudomallei* (Bzdyl et al., 2019), and *Salmonella* Typhimurium (Kumawat et al., 2020).

Brucellosis is a worldwide zoonotic disease caused by the intracellular bacterial pathogen, *Brucella* spp. *Brucella* spp. are Gram-negative bacteria that belongs to the α-2 group of *Proteobacteria*, a bacterial group characterized for living in close association with eukaryotic hosts such as plants or mammals (Corbel, 1997). *Brucella* infection causes abortion and sterility in animals, and undulating fever and debilitating disorders in humans, resulting in a serious public health problem and economic losses (de Figueiredo et al., 2015). *Brucella* virulence relies on its ability to adapt to an intracellular lifestyle within the host cells. To gain insight into the molecular mechanisms involved in intracellular adaptation and virulence of *Brucella*, we performed a comparative proteome analysis of *Brucella* grown in culture media or recovered from *Brucella* infected macrophages using two complementary technologies: 2D gel (Roset et al., 2013) and iTraq isobaric tag (Roset et al., 2017). We demonstrated through 2D gel analysis, that, upon intracellular localization, *B. abortus* over-expresses two PPIases (BAB1_1117 and BAB1_1118), belonging to the cyclophilin family (COG0652), referred to as CypB and CypA, respectively. Analysis of their function by mutagenesis and subsequent characterization showed that they are involved in stress adaptation, intracellular survival, and *Brucella* virulence (Roset et al., 2013). In this report, we characterized CypA and CypB from a biochemical and functional standpoint exploring the role of the cyclophilins in *Brucella*-host cell interaction.

## Materials and methods

### Bacterial strains and growth conditions

Bacterial strains and plasmids used are shown in Table 1. *Escherichia coli* strains were grown in Luria-Bertani (LB) media at 37°C on a rotatory shaker (250 rpm) or in LB agar for 16–24 h. *Brucella abortus* strains were grown in tryptic soy agar (TSA) or tryptic soy broth (TSB) media at 37°C on a rotatory shaker (250 rpm) for 16–24 h. When necessary, media were supplemented with the following antibiotics: kanamycin (km), 50 μg/ml, ampicillin 100 μg/ml, or nalidixic acid 5 μg/ml. Experiments involving live *Brucella* were performed in a Biosafety level 3 (BSL3) facility at the University of San Martín, Buenos Aires, Argentina.

**Table 1:** Bacterial strains and plasmids used in this study.

Strain or plasmid	Genotype or phenotype	Reference or source
Strains		
*Escherichia coli*		
BL21 DE3	F- ompT hsdSB (rBmB-) gal dcm (DE3)	Stratagen
TOP10	F-*mcrA* Δ(mrr-hsdRMS-mcrBC) Φ80*lac*ZΔM15-Δ *lac*X74 *rec*A1 *ara*D139Δ(*araleu*)7697 *gal*U *gal*K *rps*L (StrR) *end*A1 *nup*G	Invitrogen
DH5α F’IQ	F´ Φ80d*lacZ*ΔM15 Δ(*lacZYA-argF*) *U169 deoR recA1 endA1 hsdR17*(r_K_^+^ m_K_^+^) *phoA^+^ supE44* λ^−^ *thi-1 gyrA96 relA1*/F´ *proAB^+^ lacI*^q^*Z*ΔM15 *zzf*::Tn*5* (Km^r^)	Invitrogen
S17.1 (λ pir)	λ lysogenic S17-1 derivative producing π protein for replication of plasmids carrying oriR6K, Nal^S^	Herrero et al. (1990)
*Brucella abortus*		
Wild type 2,308	Wild, smooth, virulent strain, Nal^r^	Laboratory stock
2,308 (p*fcypB*)	*B. abortus* 2,308 with plasmid p*fcypB*, Amp^r^	This study
2,308 (p*fcypB^R59A/F64A^*)	*B. abortus* 2,308 with plasmid p*fcypB^R59A/F64A^*, Amp^r^	This study
Δ*cypAB* mutant	*B. abortus* 2,308 double mutant by deletion of the *cypA* and *cypB* genes	Roset et al. (2013)
Δ*cypAB(*p*cypAf*)	*B. abortus* Δ*cypAB* mutant with plasmid p*cypAf*, Amp^r^	This study
Δ*cypAB(*p*fcypB*)	*B. abortus* Δ*cypAB* mutant with plasmid p*fcypB*, Amp^r^	This study
Δ*cypAB(*p*fcypB^R59A/F64A^*)	*B. abortus* Δ*cypAB* mutant with plasmid p*fcypB^R59A/F64A^*, Amp^r^	This study
Δ*cypAB(*p*fcypB^C128M^*)	*B. abortus* Δ*cypAB* mutant with plasmid p*fcypB^C128M^*, Amp^r^	This study
Plasmids		
pET-28a(+)	Bacterial cloning vector with T7lac promoter that carries in the N-terminal a His-Tag / thrombin / T7-Tag configuration, an optional His-Tag sequence in the C-terminal and kanamycin resistance gene.	EMD Biosciences
pET-28-*cypA*	510 pb *BamH*I/*Xho*I fragment containing the *B. abortus* 2,308 *cypA* gene starting at position 82 bp, cloned into pET-28a(+), Km^r^.	This study
pET-28-*cypB*	492 pb *BamH*I/*Xho*I fragment containing the *B. abortus* 2,308 *cypB* gene starting at position 37 bp, cloned into pET-28a(+), Km^r^.	This study
pET-28-*cypB^R59A/F64A^*	492 pb *BamH*I/*Xho*I fragment containing the *B. abortus* 2,308 *cypB^R55A/F60A^* mutant gene starting at position 37 bp, cloned into pET-28a(+), Km^r^.	This study
pET-28-*cypB^W134F^*	492 pb *BamH*I/*Xho*I fragment containing the *B. abortus* 2,308 *cypB^W134F^* mutant gene starting at position 37 bp, cloned into pET-28a(+), Km^r^	This study
pET-28-*cypB^C128M^*	492 pb *BamH*I/*Xho*I fragment containing the *B. abortus* 2,308 *cypB^C128M^* mutant gene starting at position 37 bp, cloned into pET-28a(+), Km^r^.	This study
pET-28-*cypB*(*L2cypA*)	534 bp *Nhe*I/*Xho*I fragment containing the *B. abortus* 2,308 *cypB* gene starting at position 37 bp, where loop-2 from *cypB* (289–294 bp) was replaced by loop-2 of *cypA* (310–336 bp), cloned into pET-28a(+), Km^r^.	This study
pET-28-*cypB*(*L3cypA*)	525 bp *Nhe*I/*Xho*I fragment containing the *B. abortus* 2,308 *cypB* gene starting at position 37 bp, where loop-3 from *cypB* (478–489 bp) was replaced by loop-3 of *cypA* (523–546 bp), cloned into pET-28a(+), Km^r^.	This study
pET-28-*cypB*(*L2-L3cypA*)	534 bp *Nhe*I/*Xho*I fragment containing the *B. abortus* 2,308 *cypB* gene starting at position 37 bp, where loop-2 (289–294 bp) and loop-3 from *cypB* (478–489 bp) were replaced by loop-2 (310–336 bp) and loop-3 (523–546 bp) of *cypA* respectively, cloned into pET-28a(+), Km^r^.	This study
pET-28-*cypA*(*L1cypB*)	552 bp *Nhe*I/*Xho*I fragment containing the *B. abortus* 2,308 *cypA* gene starting at position 82 bp, where loop-1 from *cypA* (268–297 bp) was replaced by loop-1 of *cypB* (223–276 bp), cloned into pET-28a(+), Km^r^.	This study
p*cypB^R55A/F60A^*	*B. abortus cypB^K55A/F60A^* gene cloned into pDK51, Amp^r^	Roset et al. (2013)
pET-21-eGFP	Derived from pET21b, expresses eGFP fusion protein, Km^r^.	Fina Martin et al. (2019)
pDCyaA	Cloning vector for C-terminus fusion to CyaA under bcsp31 gene promoter, Amp^r^	Marchesini et al. (2011)
p*cypAf*	657-bp *BamH*I/*Sac*II synthetic fragment containing full-length *B. abortus cypA* gene and 3flag, cloned into pDCyaA, Amp^r^	This study
p*fcypB*	0.6-kb *BamH*I/*Sac*II synthetic fragment containing 3flag and the *B. abortus 2,308* full-length *cypB* gene, cloned into pDCyaA, Amp^r^	This study
p*fcypB^R59A/F64A^*	0.6-kb *BamH*I/*Sac*II synthetic fragment containing 3flag and the *B. abortus 2,308* full-length *cypB* gene where Arg^59^ and Phe^64^ were replaced by Ala, cloned into pDCyaA, Amp^r^	This study
p*fcypB^C128M^*	0.6-kb *BamH*I/*Sac*II synthetic fragment containing 3flag and the *B. abortus 2,308* full-length *cypB* gene where Cys^128^ was replaced by Met, cloned into pDCyaA, Amp^r^	This study

Amp^r^, ampicillin resistance; Nal^r^, nalidixic acid resistance; Km^r^, kanamycin resistance.

### Cloning

*cypB* and *cypB^R59A/F64A^* genes were amplified from *Brucella* genomic DNA or p*cypB^R55A/F60A^* plasmid, respectively, by PCR using the oligonucleotides (pFWCypBBamHI CGGGATCCGACCCAGAAAATACGCTCG and pRVCypBXhoI CCCTCGAGTCAGTCGGCGGCGATACG). Amplicons were digested with *BamH*I and *Xho*I restriction enzymes and cloned in pET-28a(+) vector from Novagen.

Synthetic genes, synthesized by Gene Universal Inc. (United States), *cypA, cypB^C128M^, cypB^W134F^, cypB*(*L2cypA*), *cypB*(*L3cypA*), *cypB*(*L2-L3cypA*), and *cypA*(*L1cypB*) were cloned in pET-28a (+). Genes *cypA3flag, 3flagcypB, 3flagcypB^R59A/F64A^*, and *3flagcypB^C128M^* were cloned in pBlueScript II SK(+) and then digested with *BamH*I and *Sac*II and subcloned in pDCyaA plasmid.

Electroporation of the *E. coli* strain was performed with the Pulser-BioRad electroporator according to the manufacturer’s protocol.

### *Brucella abortus* complementation

*Brucella abortus ΔcypAB* mutant was genetically complemented by introducing p*3flagcypA*, p*3flagcypB*, p*3flagcypB^R59A/F64A^*, and p*3flagcypB^C128M^* plasmids by biparental mating using *E. coli* S17.1 as donor strain (Ditta et al., 1980). *Brucella* complemented strains were selected in ampicillin and nalidixic acid TSA plates, and the presence of different cyclophilins were confirmed by PCR and Western blot analysis (anti-CypA and anti-CypB antibodies).

*Brucella abortus* 2,308 was genetically transformed by introducing p*3flagcypB* or p*3flagcypB^R59A/F64A^* plasmids by biparental mating using *E. coli* S17.1 as donor strain (Ditta et al., 1980). *Brucella* transformed strains were selected in ampicillin and nalidixic acid TSA plates, and the presence of different cyclophilins were confirmed by PCR and Western blot analysis (anti-3FLAG antibody).

### Purification of recombinant proteins

His-tagged recombinant proteins (CypA, CypB, CypB^R59A/F64A^, CypB^C128M^, CypB^W134F^ CypA(L1CypB), CypB(L2CypA), CypB(L3CypA), CypB(L2-L3CypA), and GFP) were expressed in *E. coli* and purified using nickel affinity chromatography. Briefly, *E. coli* strains were grown at 37°C at 200 rpm and the expression was induced with 0.1 mM IPTG at A600 = 0.5. Two hours post-induction cells were harvested at 7000 X *g* and lysed by sonication. Recombinant proteins were purified from soluble fractions with the HisTrap™ HP column (GE Healthcare). Elution was performed with an imidazole gradient (20 to 500 mM). Fractions with recombinant proteins were dialyzed and quantified with NanoDrop One Microvolume UV–Vis Spectrophotometer (Thermo Fischer). Expression was confirmed by Western blotting. The predicted molecular weight of the recombinant proteins are: 21.5 kDa (CypA), 21.1 kDa (CypB), 21.0 kDa (CypB^R59A/F64A^), 20.2 kDa (CypB^C128M^), 20.1 kDa (CypB^W134F^), 21.0 kDa (CypA(L1CypB)), 20.9 kDa (CypB(L2CypA)), 20.5 kDa (CypB(L3CypA)), 21.3 kDa (CypB(L2-L3CypA)), and 46.6 kDa (GFP).

### Protein analysis

Protein samples were suspended in cracking buffer (2% SDS, 10% Glycerol, 60 mM Tris-Cl pH 6.8, 0.01% Bromophenol Blue, and 100 mM DTT) and incubated for 5 min at 100°C. Protein electrophoresis was performed at 120 V on a 12% SDS-PAGE gel. Gels were stained in Coomassie-Blue solution (20% methanol, 10% acetic acid).

For Western Blot analysis, proteins were transferred to a nitrocellulose membrane (Immobilon – Merck Millipore Ltd) for 55 min at 15 V using a semi-dry electroblotting transfer unit (Bio-Rad, Hercules, CA, USA). Membranes were incubated for 1 h with blocking buffer (1% dry skim milk, 0.1% Tween in PBS) and then incubated for 1 h with primary antibody diluted in blocking buffer (1/500). After washing with PBS-0.1%Tween, membranes were incubated for 1 h with secondary antibody labeled with IRDye fluorophores (LI-COR, Lincoln, NE, United States) diluted in blocking buffer (1/20,000). Finally, the membranes were scanned using the Odyssey Imaging System (LI-COR).

Proteins were quantified with the UV–Vis NanoDrop One spectrometer (Thermo Scientific).

### Antibodies generation

BALB/c mice were immunized intraperitoneally with a volume of 200 μl containing 10 μg of the different purified recombinant proteins (CypA or CypB) using aluminum hydroxide as an adjuvant. Boosters with 5 μg of protein were further performed at 2 and 4 weeks. One week after the last immunization, the mice were bled, and the serum was stored at −20°C for later use.

### PPIase activity measurement

Determination of the PPIase activity of recombinant cyclophilins was performed as described (Mares et al., 2011). Briefly, a 5 μM acid denatured Green Fluorescent Protein (GFP) solution was prepared by diluting 10 μM GFP solution in denaturation buffer (150 mM NaCl, 50 mM Tris–HCl, pH 7.5) with an equal volume of 125 mM HCl solution. The mixture was incubated for 1 min at room temperature verifying the denaturation with fluorescence measurements. Then, the 2.5 μM denatured GFP solution was diluted 1:100 in refolding buffer (25 mM MgCl_2_, 100 mM KCl, 50 mM Tris–HCl, pH 7.5) in the absence or presence of cyclophilins in different concentrations. The reaction was carried out in a final volume of 200 μl at room temperature measuring the fluorescence for 20 min on the FilterMax F5 spectrometer at 485 nm excitation and 538 nm emission wavelengths.

PPIase inhibition assay was performed as it was described for activity measurement but with the addition of different concentrations of CsA (0, 5, 10, 15, and 20 μM) to the reaction mix in the presence of 2 μM of cyclophilin.

To estimate change percentage in GFP refolding assay, fluorescence value corresponding to 15-min reaction was compared. Signal corresponding to the basal control (spontaneous refolding of GFP) was considered as 0% of PPIase activity. PPIase activity of CypB (that was the highest activity in our assay) was selected as 100%.

### Chaperone activity assay: residual activity of denatured *Nde*I

The optimal denaturation temperature for the restriction enzyme *Nde*I was determined. For this, 1 U of enzyme in NEB 3.1 buffer was heated at different temperatures in the range of interest for 20 min. Then, 150 ng of pET28a (+) plasmid were added and incubated for 1 h at 37°C to digest. Restriction digestion of pET28a (+) plasmid was analyzed by 1% agarose gel electrophoresis.

To analyze the chaperone activity of the recombinant proteins, the method already described was adapted (Pandey et al., 2016). Briefly, 1, 2.5, and 5 μg of CypA, CypB, CypB^R59A/F64A^, CypB^C128M^, or 5 μg of BSA (control) were mixed with 1 U of enzyme *Nde*I in NEB 3.1 buffer and heated at 53.8°C for 20 min (optimal denaturation temperature). The residual *Nde*I activity was measured by digesting 150 ng of pET28a (+) plasmid for 1 h at 37°C. The result of the digestion was analyzed by electrophoresis on 1% agarose gel, stained with ethidium bromide, and subsequent UV visualization.

### Cell culture and infection assay

HeLa cells were maintained at 37°C in a 5% CO2 atmosphere in Dulbecco modified Eagle medium (DMEM) supplemented with 5% fetal bovine serum and streptomycin (50 μg/ml)-penicillin (50 U/ml). 5 × 10^4^ cells per well were seeded on 24-well plates and kept for 24 h in antibiotic-free DMEM. Infection with *B. abortus* was carried out with a multiplicity of infection (MOI) of 1,000: 1.

First, cells were incubated for 60 min with the bacteria. Then, to eliminate non-internalized bacteria, wells were washed five times with phosphate-buffered saline (PBS) and incubated with a fresh medium supplemented with 50 μg/ml gentamicin and 100 μg/ml streptomycin. Finally, infected cells at 4 h post-infection were washed with PBS five times and lysed with 500 μl 0.1% Triton X-100. Intracellular CFU was determined by plating serial dilutions in TSA with the corresponding antibiotics.

### Sensitivity to deoxycholate assay (DOC)

*Brucella abortus* cultures were adjusted to a standardized optical density at *λ* = 600 nm (OD_600_) and suspended in 1 ml of PBS. Immediately, cultures were serially diluted in PBS and plated in TSA plates containing 1,000 μg/ml deoxycholate. Plates were incubated for 3 days at 37°C.

### Mouse infection assay

Before inoculation, 0.1 ml of 10% sodium bicarbonate was administrated to groups of five female BALB/c mice. Oral infection was performed with a volume of 200 μl containing 10^9^ CFU of *Brucella*. Mice were euthanized at 6 weeks after infection and bacteria were recovered from the spleens. Spleens were homogenized in 2 ml of PBS, and serial dilutions were plated on TSA.

### ELISA assay

Enzyme-linked immunosorbent assay was performed as published (Uriza et al., 2020). Briefly, wells were coated with 125 ng of recombinant proteins in 50 μl of buffer (0.5 M carbonate–bicarbonate pH 9.6). Wells were washed four times (0.1% Tween 20 in PBS buffer). Then, 50 μl of primary antibodies α-CypA or α-CypB per well (1:500) were incubated for 1 h. After that, wells were washed again four times and incubated for 1 h with HRP-anti-mouse IgG (1, 1,000). Finally, wells were washed and incubated for 10 min with 50 μl of substrate solution containing 3, 3′,5,5’ Tetramethylbenzidine (Sigma) and 50 μl of stopping solution for 10 min. Absorbance was measured in the FilterMax F5 Microplate Reader at 450 nm.

### Protein structure modeling

Protein structure modeling and comparison between CypA and CypB protein structures were conducted by SWISS-MODEL^1^ and UCSF Chimera 1.14 software, respectively (Meng et al., 2006).

### Sequences alignment

Sequences alignment comparison of CypA and CypB was done with UCSF Chimera 1.14 (Meng et al., 2006).

### Statistical analysis

GraphPad Prism 5 software was used to perform graphs and statistical analyses. One-way analysis of variance (ANOVA) with Bonferroni post-hoc test and Two-way ANOVA-Tukey’s multiple comparison test (within each row, compare columns) were used to analyze statistical significance.

## Results

### *Brucella*’s Cyps have in common a typical core domain of the cyclophilin fold but present different structural characteristics

**Figure 1:**
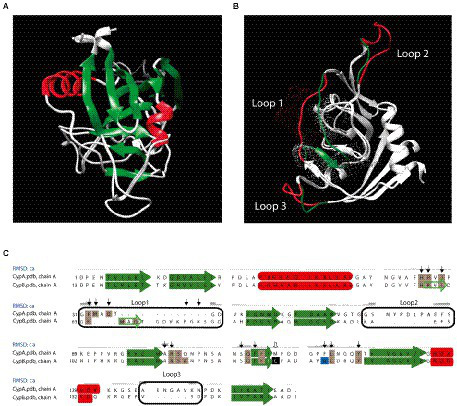
Comparison of the three-dimensional structural model of *Brucella abortus* cyclophilins. **(A)** Comparison of the three-dimensional structural model for *B. abortus* cyclophilins CypA and CypB. Swiss-model and Chimera 1.14 programs were used for the model and to compare three-dimensional structural, respectively. The Swiss-model program selected the structure of AquaCyp293 (PDB ID: 5ex2.1A) as a template for CypA and CypB. The predicted general protein structure consists of eight antiparallel beta sheets (green) and two alpha-helices (red). **(B)** Differential loops detected between CypA and CypB were indicated in red and green, respectively. Dot clouds highlighted the PPIase active site areas **(C)** Sequence alignment of *B. abortus* cyclophilins. Amino acid residues involved in cyclosporin (CsA) binding are indicated by black arrows, and those involved in peptidyl-prolyl *cis*/*trans* isomerase (PPIase) activity are highlighted in gray. Cysteine is indicated by a white arrow and highlighted in black and conserved tryptophan is highlighted in light blue. Secondary structures are indicated in green arrows (beta sheets) and red boxes (alpha-helices). Differential peptide loops are marked with black boxes. The alignment was performed with the Chimera 1.4 program.

**Figure 2:**
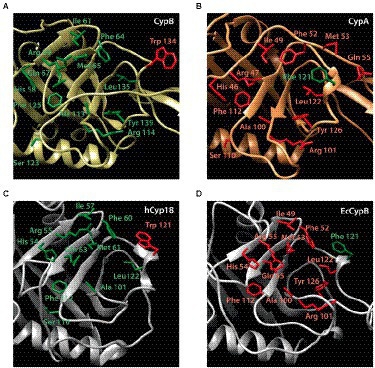
Active site structure of *Brucella abortus* cyclophilins CypB **(A)** and CypA **(B)** [in comparison with hCyp18 (2cpl.pdb) **(C)** and EcCypB (1Lop.pdb) **(D)**]. Residues that contribute to the active site of the cyclophilin family are labeled and shown in stick representation. The tryptophan involved in CsA inhibition is highlighted in red for CypB (Trp^134^) and hCyp18 (Trp^121^). Phe^121^ is highlighted in green for CypA and EcCypB.

A three-dimensional structural model of the cyclophilins CypA and CypB from *B. abortus* was built, showing that both Cyps have in common several secondary structural features. As shown in Figure 1A, the cyclophilin domain of both CypA and CypB share eight stranded antiparallel β-barrel with two α-helix covering the top and the bottom of the barrel, which is consistent with other structures from the cyclophilin family, such as the human PpiA and the *E. coli* EcCypB (Ke et al., 1991; Ke, 1992; Edwards et al., 1997). However, the 3D structure modeling of both cyclophilins was also able to reveal some differences in their structure over three peptide loops (Figures 1B,C). As shown in Figures 1B,C and Figure 2, catalytic active sites of both Cyps are completely conserved suggesting that both cyclophilins can potentially recognize similar protein substrates. Sequence alignment and 3D models also highlighted the presence of a critical tryptophan at position 134 of the CypB sequence (Trp^134^) which is absent in *Brucella* CypA and replaced by a phenylalanine in the equivalent position (Phe^121^). As shown in Figure 2, this critical tryptophan is also present within the active site of the human hCyp18 and absent in the *E. coli* EcCypB, where it is also replaced by phenylalanine. Of particular interest, it has been reported that this conserved tryptophan is critical for the interaction with the immunosuppressor compound cyclosporin A (CsA) in all the described eukaryotic Cyps (Bossard et al., 1991; Liu et al., 1991). Differently, in cyclophilins derived from gram negative-bacteria, this critical tryptophan is absent, a modification that correlates with the CsA-insensitivity observed for these Cyps (Liu and Walsh, 1990; Liu et al., 1991). Another feature, which is characteristic of eukaryotic cyclophilins (eCyps), is the presence of cysteines along their amino acid sequence (Liu et al., 1990) that in some cases have been reported to modulate the PPIase activity (Motohashi et al., 2003; Gourlay et al., 2007). Interestingly, *Brucella* CypB has a single cysteine in its sequence which is not conserved in *Brucella* CypA (Figure 1C).

**Figure 3:**
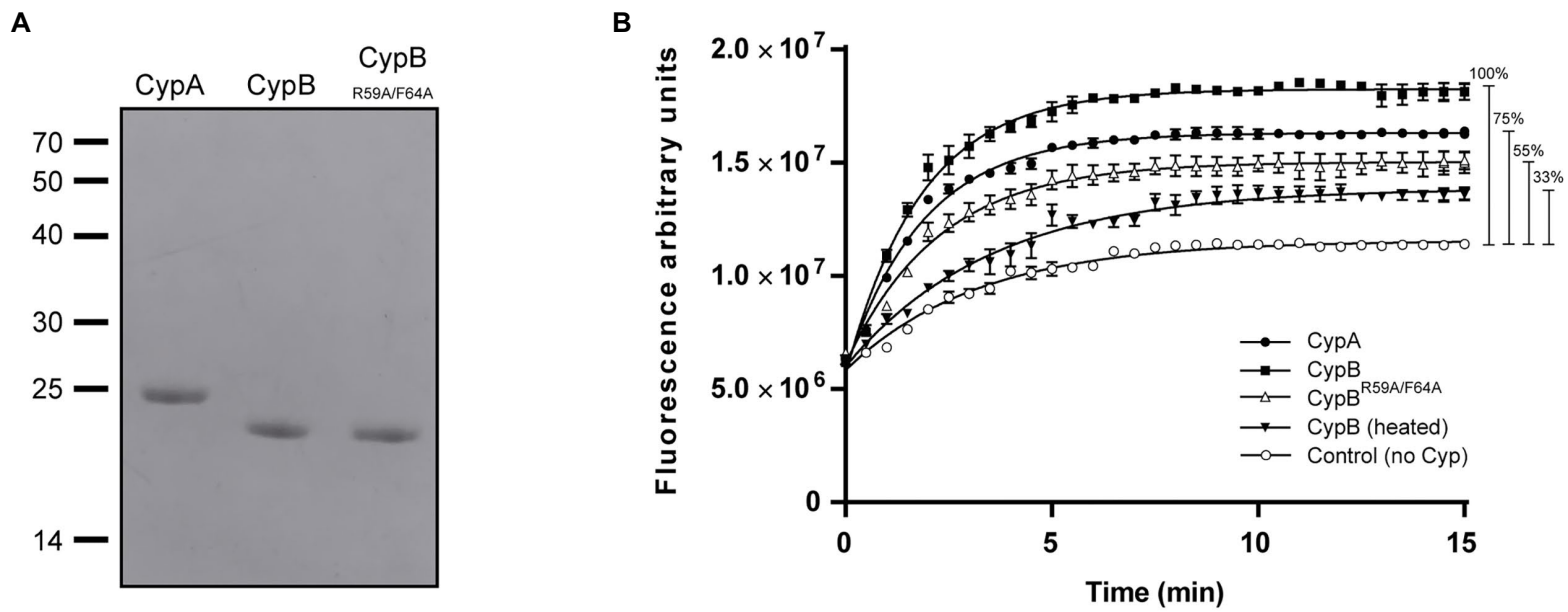
PPIase activity determination of recombinant CypA, CypB, and CypB^R59A/F64A^ proteins. **(A)** SDS-PAGE of purified recombinant cyclophilins stained with Coomassie blue. **(B)** GFP PPIase activity assay with 2 μM of CypA, CypB, and with the point mutant in the active site CypB^R59A/F64A^. The figure shows the mean and standard deviation of the triplicate experiment and is representative of three independent experiments. Two-way ANOVA and Tukey’s multiple comparison test (within each row, compare columns) were used to analyze the statistical significance of the results, ****p < 0.0001.

Considering that differences between *Brucella* CypA and CypB might reflect diverse physiological functions we decided to characterize both Cyps from a biochemical and functional standpoint. With that in mind, genes encoding CypA or CypB were recombinantly expressed in *E. coli* BL21 (DE3) and further purified using Ni-NTA chromatography as described in Materials and Methods (Figure 3A). These recombinant proteins were used for the characterization of biochemical activities.

### *Brucella* cyclophilins CypA and CypB exhibit PPIase activity, but only CypB is inhibited By cyclosporin A

The *B. abortus* cyclophilins CypA and CypB present a typical isomerase domain, containing residues involved in PPIase activity (Figures 1C, 2). To confirm if *Brucella* CypA and CypB are functional PPIases, their enzymatic determination was performed with purified recombinant cyclophilins, as described by Mares et al. (2011). To test cyclophilin enzymatic activity, refolding of acid-denatured GFP was adapted for PPIase determination since proline isomerization is the limiting step in the refolding process of GFP (Andrews et al., 2007). As shown in Figure 3B, the addition of CypA or CypB resulted in a highly significant improvement (*p* < 0.0001) in the refolding of the acid-denatured GFP confirming their true enzymatic activity. Interestingly, results from the GFP refolding assay showed that CypB presented a higher rate of reaction compared with CypA that presented an activity reduction of 25% (Figure 3B). To confirm PPIase activity, a double point mutation was performed in CypB to replace two amino acids reported to be critical for PPIase activity (R59A and F64A; Zydowsky et al., 1992). The resulting recombinant protein CypB^R59A/F64A^ was expressed and purified (Figure 3A) and used in the GFP refolding assay. As shown in Figure 3B, CypB^R59A/F64A^ displayed a 45% diminished PPIase activity compared with CypB.

**Figure 4:**
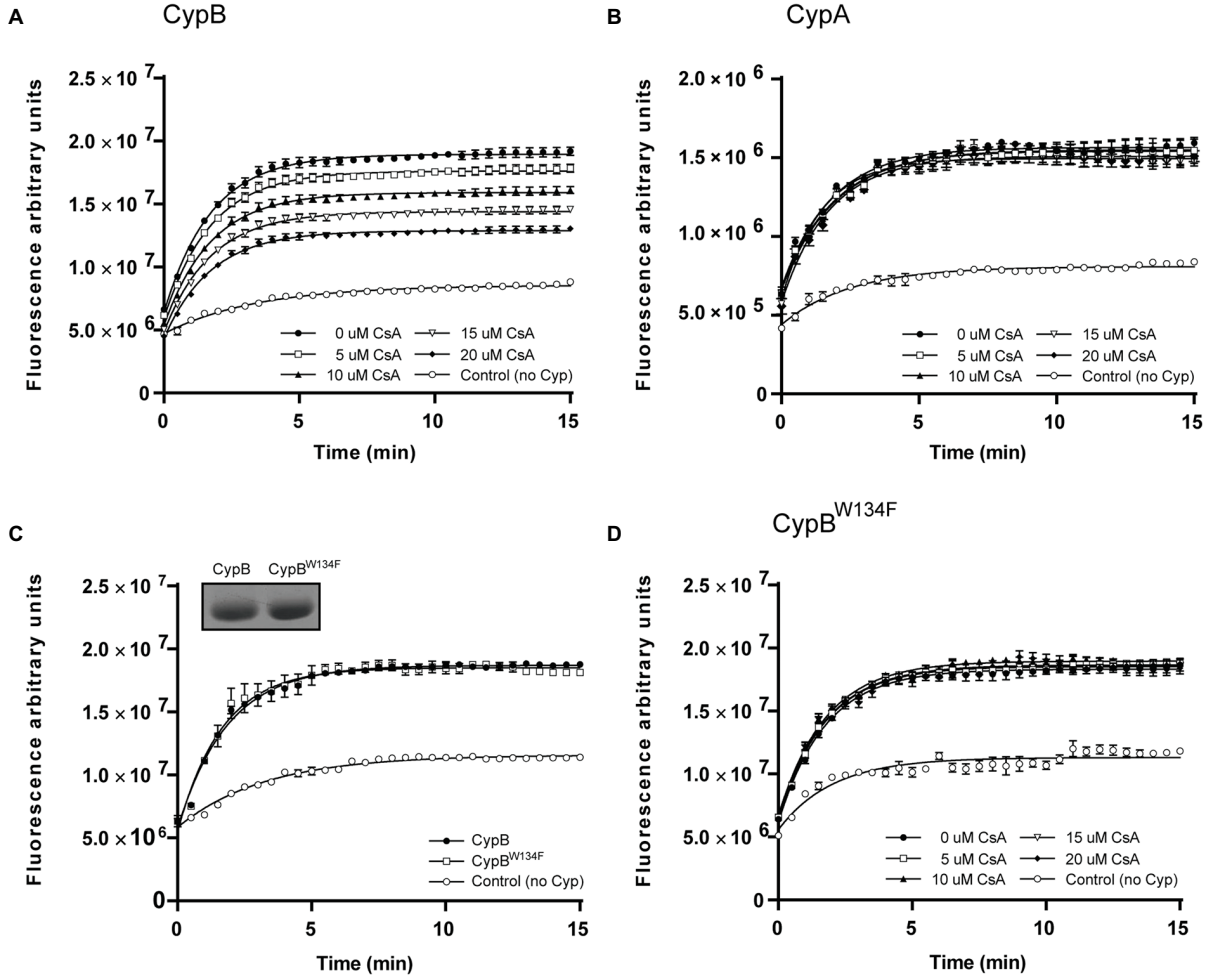
Inhibition of enzyme activity by cyclosporin A. CypB **(A)**, CypA **(B)**, or CypB^W134F^ **(D)** were preincubated with increasing concentrations of CsA and the remaining PPIase activity was analyzed with the GFP PPIase activity assay. **(C)** GFP PPIase activity assay with 2 μM of CypB^W134F^. In inset Coomassie blue of purified CypB^W134F^. The figures show the mean and standard deviation of triplicate experiments and are representative of three independent experiments. Two-way ANOVA and Tukey’s multiple comparison test (within each row, compare columns) were used to analyze the statistical significance of the results, ****p < 0.0001.

As shown in Figures 1C, 2, a comparative analysis of the amino acid sequence between CypA and CypB revealed the presence of some features in CypB which are characteristic of eukaryotic cyclophilins like the presence of a critical tryptophan (Trp^134^), an amino acid predicted to be involved in the CsA interaction (Bossard et al., 1991; Liu et al., 1991). Thus, we explored the inhibition effect of CsA on the enzymatic activity of CypA and CypB of *B. abortus*. After the preincubation with increasing concentrations of CsA, the PPIase activity was highly significantly inhibited (*p* < 0.0001) in a dose-dependent manner in the case of CypB (more than 50% inhibition with 15 μM; Figure 4A), but not in CypA (Figure 4B). CsA inhibition of CypB was abolished in the case of CypB^W134F^ mutant, where tryptophan, was replaced for phenylalanine (Figures 4C,D). Altogether, these results are in accordance with the *in-silico* prediction that CypB behaves in terms of CsA inhibition like a eukaryotic cyclophilin.

### *Brucella abortus* cyclophilins CypA and CypB protect *Nde*I from thermal inactivation

**Figure 5:**
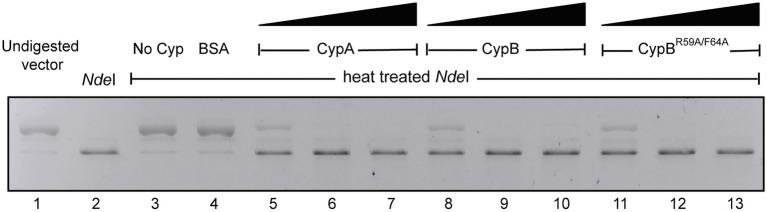
CypA and CypB of *B. abortus* prevent thermal inactivation of *Nde*I activity. Enzymatic activity of thermal-denatured *Nde*1 (53.8°C for 20 min) in the absence or presence of CypA, CypB, CypB^R59A/F64A^, or BSA as a control protein. Lane 1, uncut pET28 plasmid; lane 2, pET28 plasmid incubated with native *Nde*I; Lane 3, pET28 plasmid incubated with heat-inactivated *Nde*I; Lane 4, pET28 plasmid incubated with heat-inactivated *Nde*I in the presence of BSA (5 μg); Lane 5, 6, and 7, pET28 plasmid incubated with heat-inactivated *Nde*I in the presence of increasing concentrations of CypA protein (1, 2.5, and 5 μg); Lane 8, 9, and 10, pET28 plasmid incubated with heat-inactivated *Nde*I in the presence of increasing concentrations of CypB protein (1, 2.5, and 5 μg); Lane 11, 12, and 13, pET28 plasmid incubated with heat-inactivated *Nde*I in the presence of increasing concentrations of CypB^R59A/F64A^ protein (1, 2.5, and 5 μg). The gel is representative of three independent experiments.

It has been described that some cyclophilins, in addition to their PPIase activity, also have chaperone activity (Dimou et al., 2011; Zhang et al., 2013; Pandey et al., 2016). Therefore, we investigated whether CypA and CypB recombinant proteins of *B. abortus* were able to prevent the thermal denaturation of the restriction enzyme *Nde*I, an assay used to determine chaperone activity. The ability of *Nde*I to digest the pET-28a(+) plasmid in 1 h at 37°C after thermal denaturation (53.8°C for 20 min), was evaluated in the presence or absence of cyclophilins. As shown in Figure 5, pET-28a(+) plasmid (lane 1) when was incubated with native *Nde*I was fully digested (lane 2). In absence of cyclophilins or in presence of BSA as a control, *Nde*I enzyme activity was completely inactivated by the heat treatment and therefore was not able to cut and linearize the pET-28a(+) plasmid (Figure 5, lane 3–4). Interestingly, the addition of increasing concentrations of CypA or CypB during the heat treatment protected *Nde*I from thermal inactivation (Figure 5, lanes 5–10). These results demonstrated that both cyclophilins have chaperone-like activity. To determine if this chaperone-like activity was dependent on PPIase activity, the double point mutant CypB^R59A/F64A^ was also analyzed. As shown in Figure 5, lanes 11–13, CypB^R59A/F64A^ protects against thermal denaturation to the same extent as CypB, indicating that chaperone-like activity is independent of the PPIase activity.

### *Brucella abortus* cyclophilins CypA and CypB present different immunodominant loops in their structure

**Figure 6:**
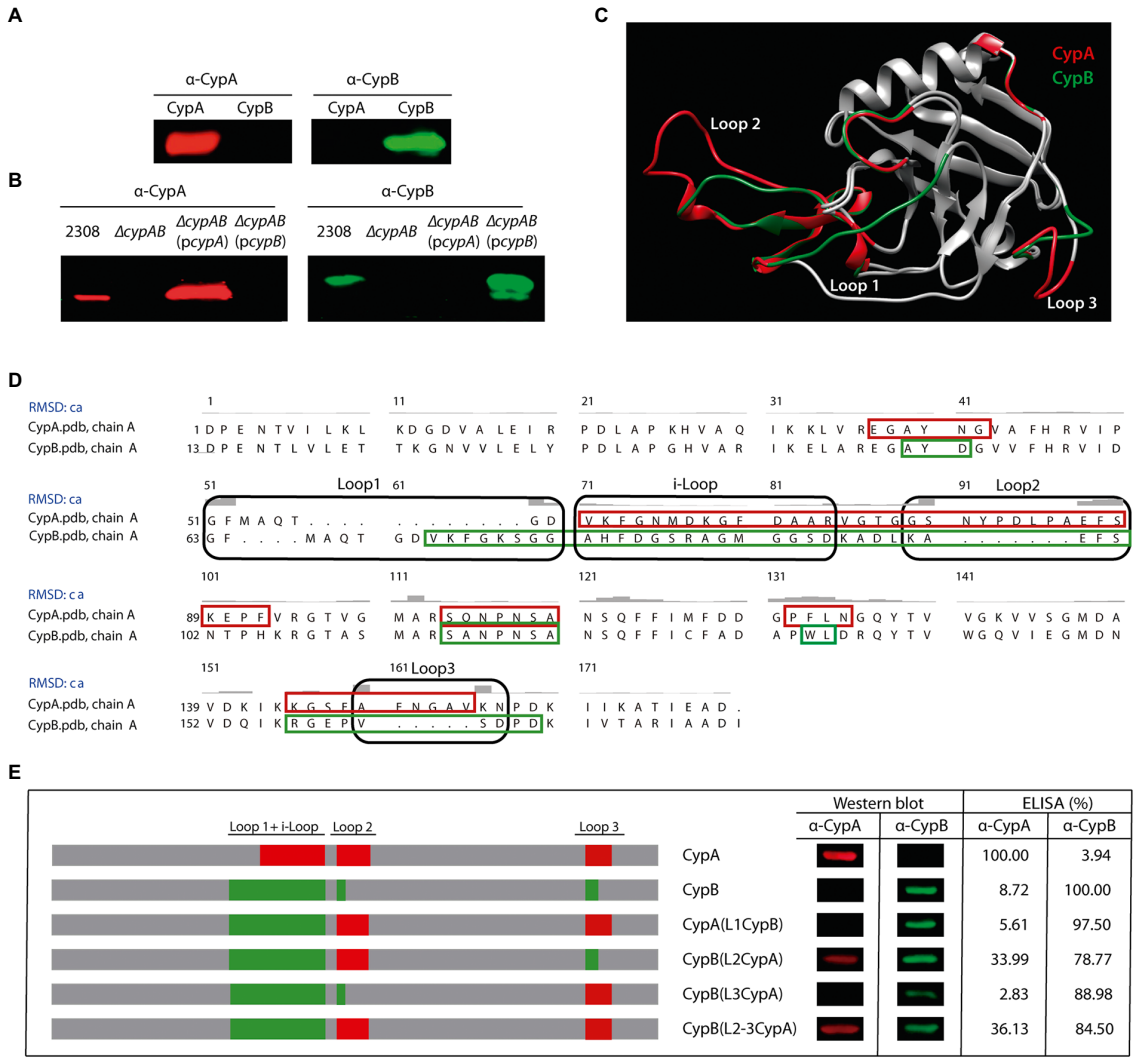
Differential immunodominant loops between CypA and CypB. Western blot analysis of **(A)** CypA and CypB recombinant proteins and **(B)** different *Brucella* whole-cell lysates probed with mouse antibodies produced against CypA or CypB. **(C)** Overlap of the three-dimensional structural model for *B. abortus* cyclophilins CypA and CypB. Swiss-model and Chimera 1.14 programs were used for modeling and the comparison of the predicted three-dimensional structure, respectively. The BepiPred-2.0 algorithm was used to predict linear epitopes. Immunodominant epitopes of CypA and CypB are marked in red and green, respectively. **(D)** Sequence alignment of *B. abortus* cyclophilins. Loops and i-Loop are indicated in a black open box. Linear epitopes are indicated in green open boxes for CypA or red open boxes for CypB. **(E)** Western blot analysis and indirect ELISA of different recombinant chimeras of CypA and CypB were revealed with anti-CypA or anti-CypB antibodies. The results are representative of three independent experiments.

To generate molecular tools for this research, recombinant proteins CypA and CypB were used as antigens to produce antibodies in mice. As shown in Figure 6A, when evaluating mouse sera, we found that antibodies raised against CypA were not able to recognize the recombinant CypB and vice versa. As shown in Figure 6B, the same was also observed in whole-cell lysates of *B. abortus* 2,308, *B. abortus* ∆*cypAB (pcypA)*, and *B. abortus* ∆*cypAB(pcypB)*. As expected, no signal was detected for the whole-cell lysate of *B. abortus* ∆*cypAB* mutant either with α-CypA or α-CypB antibodies. These results are remarkable because, although CypA and CypB share a 63% of identity in the amino acid sequence, the immune system still was able to reveal structural differences existing between both cyclophilins. As shown in Figure 6C,D, these antigenic differences can be potentially mapped on three differential amino acid loops. Interestingly, analysis of CypA and CypB sequences by the BepiPred-2.0 algorithm predicted a series of differential linear epitopes that can be located within these differential loops (Figures 6C,D). To pinpoint which peptide loop is contributing to the antibody differential recognition, an experimental approach of swapping loops between CypA and CypB was performed (Figure 6E). Based on the 3D structure prediction, it was observed that formation of loop-1 was determined by the interloop (i-Loop) region (Figures 6D,E). Consequently, we swapped loop1 + i-Loop either of CypA or CypB. To analyze linear and conformational epitopes of CypA, CypB, and their derived chimeras, Western blot analysis, and ELISA tests were carried out (Figure 6E). Thus, the replacement of loop-1 of CypA by the loop-1 from CypB (see CypA(L1CypB) in Figure 6E) determined the lack of recognition of α-CypA antibody and the gain of recognition of α-CypB antibody indicating that loop-1 contained a major determinant of the differential immunogenicity of *B. abortus* Cyps. In addition, loop-2 of CypA was also important for the recognition of α-CypA antibody since CypB(L2CypA) and CypB(L2-L3CypA) were recognized by α-CypA antibodies. Analysis of CypB(L3CypA) immunogenicity suggested that loop-3 of both Cyps is devoid of immunodominant epitopes. Altogether these immunological results confirmed structural differences predicted by the *in-silico* analysis between CypA and CypB.

### CypB forms homodimers that are sensitive to reduction

**Figure 7:**
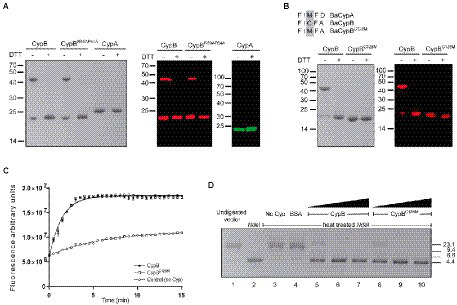
CypB is a homodimeric protein. Coomassie blue staining and Western blot analysis of protein samples incubated in the presence or absence of DTT. **(A)** CypB, CypB^R59A/F64A^, CypA, **(B)** CypB and CypB^C128M^. **(C)** GFP PPIase activity assay with 2 μM of CypB, CypB^C128M^, or without CypB. **(D)** Enzymatic activity of thermal-denatured *Nde*1 (53.8°C for 20 min) in the presence of CypB, CypB^C128M^_,_ or control protein BSA. Lane 1, uncut pET28 plasmid; Lane 2, pET28 plasmid incubated with native *Nde*I; Lane 3, pET28 plasmid incubated with heat-inactivated *Nde*I; Lane 4, pET28 plasmid incubated with heat-inactivated *Nde*I in the presence of BSA (5 μg), Lane 5, 6, and 7, pET28 plasmid incubated with heat-inactivated *Nde*I in the presence of increasing concentrations of CypB protein (1, 2.5, and 5 μg); Lane 8, 9, and 10, pET28 plasmid incubated with heat-inactivated *Nde*I in the presence of increasing concentrations of CypB^C128M^ protein (1, 2.5, and 5 μg). The results are representative of three independent experiments.

Since it was reported that PPIases can form homo-oligomers (Budiman et al., 2009; Zhang et al., 2011; Jakob et al., 2016; Polley et al., 2016) we studied the possibility that CypA and CypB were able to oligomerize. As shown in Figure 7A, when we subjected CypA or CypB to SDS-PAGE and Western blot analysis, a single protein band corresponding to the recombinant CypA or CypB was detected as expected for the monomeric form of these cyclophilins. However, when samples were treated in a non-reducing condition (without DTT in the loading sample) in addition to the monomeric band of CypB or CypB^R59A/F64A^, a larger band compatible with the formation of a homodimer of CypB or CypB^R59AF64A^ was observed (Figure 7A). Interestingly, CypA either in the presence or absence of DTT was observed like a single band, as expected for a monomeric CypA (Figure 7A). These results indicate that CypB but not CypA was able to interact to form homodimers in solution, a process that was independent of the PPIase activity.

As shown above, a comparison between both cyclophilin sequences showed that CypB has a unique cysteine residue which is absent in the CypA sequence (Figure 1C). To study the potential role of this conserved cysteine residue in dimer formation, a point mutant CypB^C128M^ was constructed, where the Cys^128^ was replaced by methionine (Figure 7B). The resulting recombinant protein CypB^C128M^ was expressed and purified and its ability to form homodimers was evaluated. As shown in Figure 7B, CypB^C128M^ was not able to form homodimers in non-reducing conditions indicating that cysteine was responsible for CypB dimerization. In addition, it was interesting to investigate if Cys^128^ was also important for PPIase or chaperone activities of CypB. As shown in Figure 7C, the PPIase activity of CypB^C128M^ was not different from CypB. In addition, results shown in Figure 7D indicated that CypB^C128M^ was also able to protect *Nde*I from thermal denaturation as efficiently as CypB. Altogether these results showed that the loss of the ability to form homodimers does not affect the *in vitro* activities of CypB analyzed in this study.

### Homodimeric CypB is important for stress survival and virulence of *Brucella abortus*

**Figure 8:**
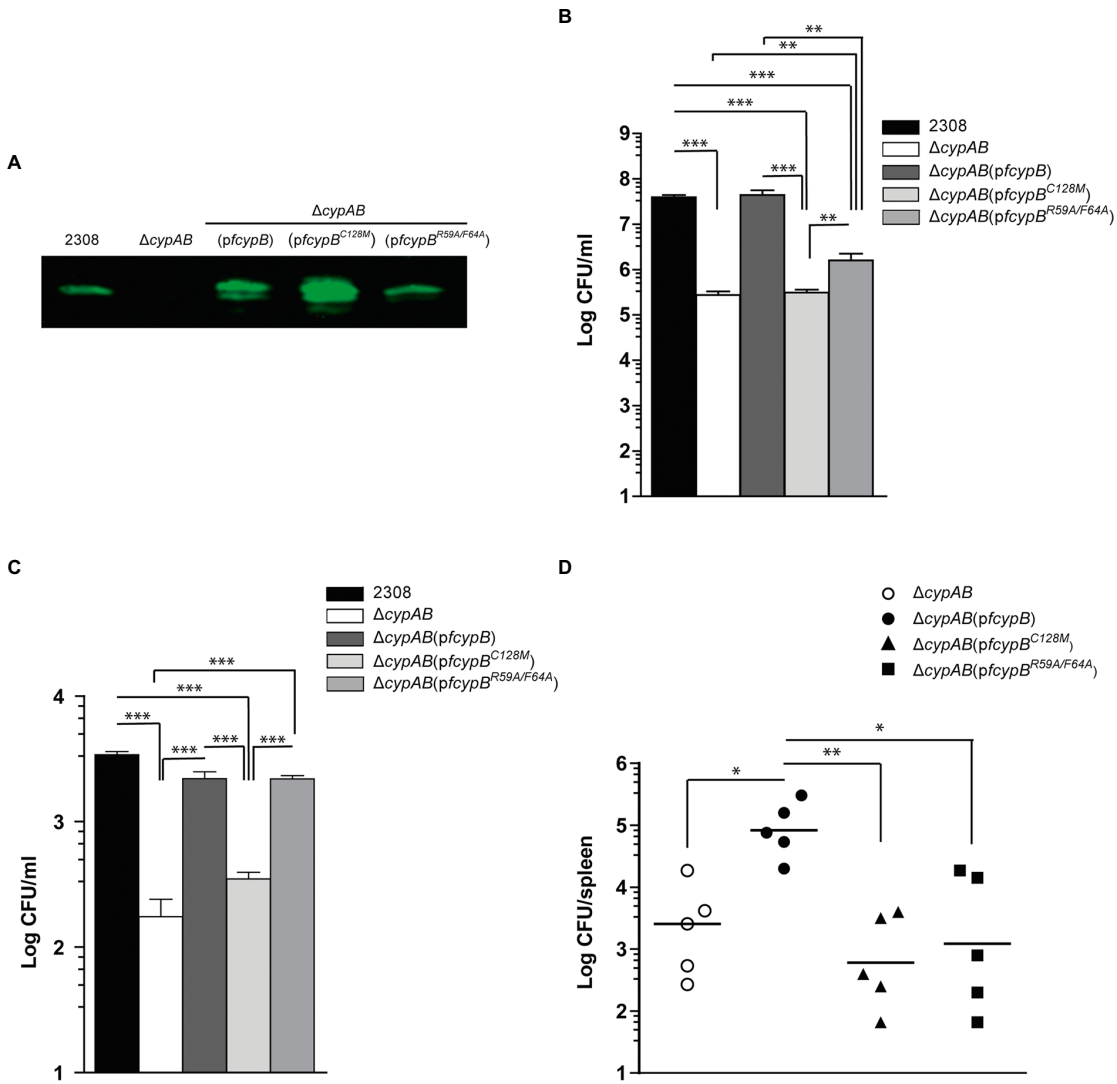
Dimeric form of CypB is required for survival and virulence of *B. abortus.* **(A)** Western blot analysis of whole-cell lysates of *B. abortus* strains expressing wild-type CypB, the monomeric form CypB^C128M^, and the active site mutant CypB^R59A/F64A^ detected by a mouse antibody against CypB. **(B)** Detergent sensitivity assay using serial dilutions of different *B. abortus* strains plated in triplicate onto TSB agar plates containing deoxycholate (DOC) (1,000 μg/ml). Plates were incubated for 72 h, and the number of CFU was scored. **(C)** Intracellular survival of the *B. abortus* strains in HeLa cells. Numbers of CFU of intracellular bacteria were determined after lysis of infected cells at 4 h post-infection. Each determination was performed in triplicate, and values are shown as the mean with its respective standard deviation and are representative of three independent experiments. **(D)** BALB/c mice were infected orally (1 × 10^9^ CFU) with different *B. abortus* strains. At 6 weeks post-infection, the numbers of CFU recovered from spleens were determined by serial dilutions and plating onto TSA. Five animals were used for each determination. One-way ANOVA and Bonferroni’s Multiple Comparison Test were used to analyze the statistical significance of the results. *, *p* < 0.05, **, *p* < 0.01, *** *p* < 0.001.

Although the loss of CypB ability to form homodimers showed no effect on the *in vitro* PPIase or chaperone activities, we explored if CypB oligomerization can affect *in vivo* functions of CypB in *B. abortus* related to stress and intracellular host-cell adaptation. For that, the plasmid p*fcypB^C128M^* was introduced into *B. abortus* ∆*cypAB* mutant strain and the stress adaptation, intracellular survival, and virulence in mice were examined (Figure 8). The expression of CypB^C128M^ protein in *B. abortus* was confirmed by Western blot analysis (Figure 8A). As described previously in Roset et al. (2013) ∆*cypAB* deletion generates a phenotype in *B. abortus* characterized by an increased sensitivity to a set of different stressors such as deoxycholate acid (DOC). As shown in Figure 8B, the plasmid p*fcypB^C128M^* was unable to complement the DOC sensitivity of *B. abortus* Δ*cypAB* mutant. These results indicated that CypB homodimer formation is necessary for *Brucella* stress adaptation.

To examine if the homodimeric formation of CypB is required for *B. abortus* intracellular survival, Hela cells were infected with *B. abortus* 2,308 wild-type strain, *B. abortus* Δ*cypAB* mutant, *B. abortus* Δ*cypAB*(p*fcypB*), *B. abortus* Δ*cypAB*(p*fcypB^R59A/F64A^*) and, *B. abortus* Δ*cypAB*(p*fcypB^C128M^*) (Figure 8C). Results showed that at 4 h post-infection *B. abortus* Δ*cypAB*(p*fcypB^C128M^*) showed a 10-fold reduction in intracellular survival, like what was observed in *B. abortus* Δ*cypAB* mutant suggesting that the dimeric form of CypB was also necessary for intracellular survival in Hela cells. Since *B. abortus* Δ*cypAB*(p*fcypB^C128M^*) was affected in intracellular adaptation it was interesting to investigate if this mutant was also affected in the mouse infection model. As shown in Figure 8D, orally infected mice had a reduced number (more than hundred-fold decrease) of *B. abortus* Δ*cypAB*(p*fcypB^C128M^*) in spleen at 6 weeks post-infection compared with those infected with *B. abortus* Δ*cypAB*(p*fcypB*) and like *B. abortus* Δ*cypAB* mutant. These results also showed that the dimeric form of *Brucella* CypB was fully required to establish a persistent infection in mice. All these results showed that homodimer formation was necessary for the *in vivo* functions of CypB.

### PPIase activity of CypB is required for full virulence of *Brucella abortus* in the mouse model

As we described previously (Roset et al., 2013), the plasmid p*cypB^R59A/F64A^* partially rescued the *B. abortus* Δ*cypAB* mutant for DOC sensitivity (Figure 8B). Intermediate results observed in complementation assays with p*cypB^R59A/F64A^* can be explained by the existence of certain residual PPIase activity in the cyclophilin mutant CypB^R59A/F64A^ (Figure 3B). This CypB^R59A/F64A^ residual activity was sufficient to complement intracellular survival of the *B. abortus* Δ*cypAB* mutant to the wild-type level (Figure 8C; Roset et al., 2013). To understand if the residual cyclophilin activity present in the mutant protein CypB^R59A/F64A^ was also sufficient to complement the *B. abortus ΔcypAB* mutant in the mouse model, an infection experiment was performed. As shown in Figure 8D, after 6 weeks post-infection *B. abortus* Δ*cypAB*(p*fcypB^R59A/F64A^*) was 66-fold less efficient in spleen colonization in BALB/c mice than *B. abortus* Δ*cypAB*(p*fcypB*) and similar to *B. abortus* Δ*cypAB* mutant. It is possible to speculate that the discrepancies observed can be explained by the different requirements of CypB activity dependent on the chosen experimental model. Thus, mouse oral infection is predicted to be the most demanding assay since *B. abortus* Δ*cypAB* mutant had to face a variety of sequential stressors when progressing in the intestinal tract (pH, bile salts, proteases, etc). Consequently, the mouse experiment highlighted the critical importance of CypB PPIase activity in *Brucella* intracellular survival and virulence.

### The *cypB^R59A/F64A^* gene functions as a dominant-negative mutant

**Figure 9:**
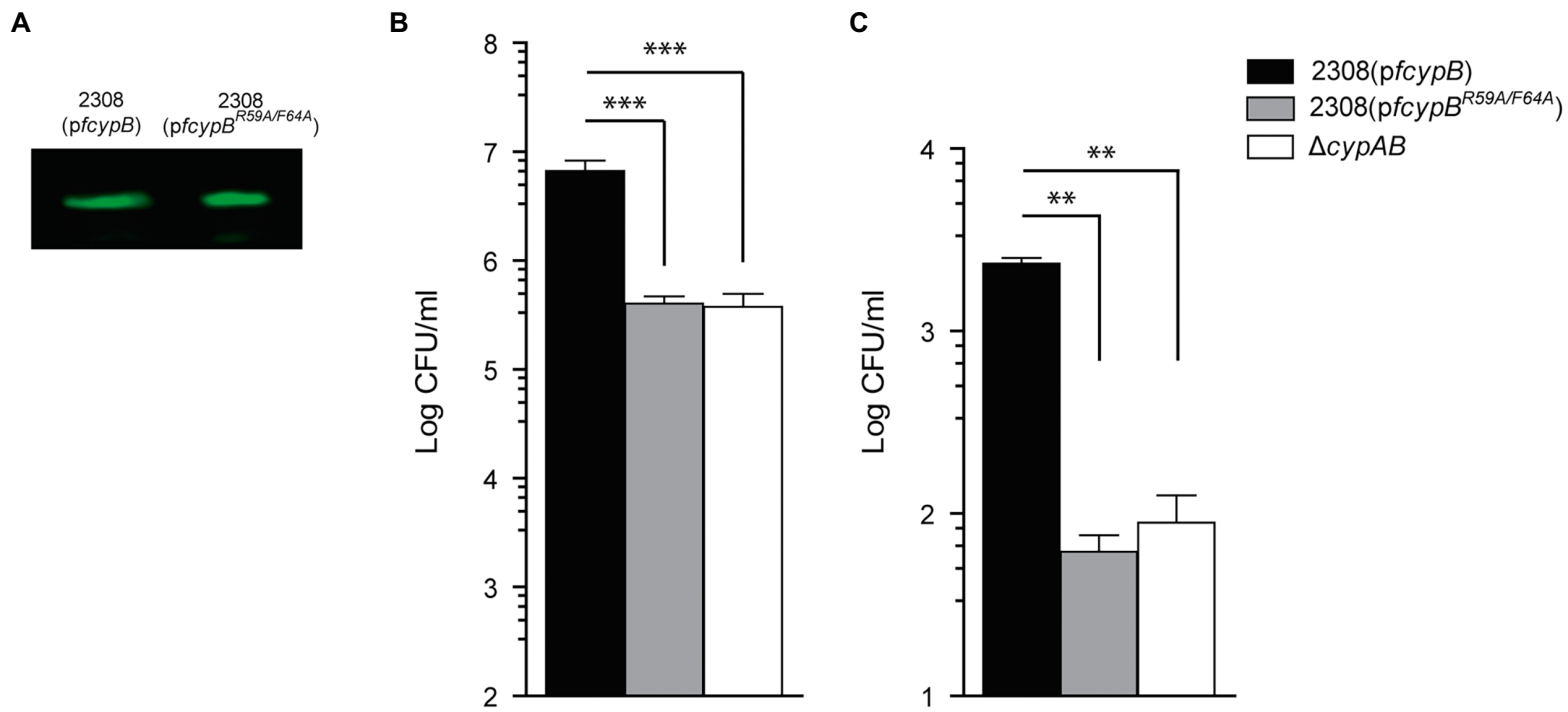
Dominant-negative mutation. **(A)** Western blot analysis of whole-cell lysates of *B. abortus* 2,308 strain expressing wild-type CypB, or CypB^R59A/F64A^ detected by an anti-flag antibody. The strains were assayed for detergent sensitivity (DOC) **(B)**, and survival in HeLa cells **(C)** as described in “Materials and methods.” One-way ANOVA and Bonferroni’s Multiple Comparison Test were used to analyze the statistical significance of the results. The results shown are representative of three independent experiments, **p < 0.01, ***p < 0.001.

To explore if the loss-of-function mutation of CypB (*cypB^R59A/F64A^*) can exert a dominant-negative effect on *B. abortus* 2,308 wild-type strain, the plasmid p*fcypB^R59AF64A^* was introduced into this strain by biparental mating and ectopic expression of CypB^R59A/F64A^ was confirmed by Western blot analysis (Figure 9A). As shown in Figure 9, the expression of CypB^R59A/F64A^ impaired the ability of *Brucella* to survive in DOC sensitivity assay (Figure 9B) and reduced 70-fold the ability to survive within HeLa cells (Figure 9C). Considering that CypB^R59A/F64A^ still can interact with CypB wild type to form dimers, these results might suggest that CypB requires dimer formation for full activity, having both monomers a fully intact active site.

## Discussion

Cyclophilins are a family of highly conserved enzymes that catalyze the process of cis-trans isomerization of the Xaa-proline bonds, which is the rate-limiting step in protein folding. This activity is critical for many biological processes including bacterial virulence (Dimou et al., 2017). We have previously reported that *B. abortus* has two cyclophilins, named cyclophilin A (CypA) and cyclophilin B (CypB) that are upregulated within the intraphagosomal replicative niche during *B. abortus* infection (Roset et al., 2013). In addition, we have also demonstrated that both cyclophilins play an important role in stress adaptation, intracellular survival, and virulence. Interestingly, defective phenotypes for stress and intracellular survival can be complemented either with CypA or CypB alone, suggesting that both Cyps show certain redundancy in their functions (Roset et al., 2013). In this report, we have also identified a group of differential features by comparing CypA with CypB. For instance, although both *B. abortus* cyclophilins share a conserved secondary structure, we have identified structural differences over three immunodominant loops identified by an *in-silico* approach and by different immunoassays. Cyclophilins are enzymes that can interact with different protein targets to help them in their folding process allowing them to acquire their functional protein structure. It would be interesting to hypothesize if differences observed in structure and antigenicity of CypA and CypB might also be reflecting differences in their preferred protein targets that can reveal novel functions for these cyclophilins.

We demonstrated here that unlike CypA, CypB presents a Trp^134^ which is involved in CsA inhibition, like in all characterized Cyps from eukaryotic origin (Bossard et al., 1991; Liu et al., 1991). On the other hand, CypA shares homology with Cyps of Gram-negative bacteria, presenting a replacement of the Trp^134^ by a Phe residue, a modification that correlates with CsA insensitivity observed for this kind of Cyps (Liu and Walsh, 1990; Liu et al., 1991). Interestingly, the replacement of Trp^134^ in CypB (CypB^W134F^) does not modify the PPIase activity either in the presence or absence of CsA indicating that Trp^134^ is not required for peptidyl-prolyl cis-trans isomerization. As mentioned above, another characteristic feature that has been described in eukaryotic cyclophilins is the presence of cysteines in their sequences (Liu et al., 1990; Motohashi et al., 2003; Gourlay et al., 2007). *Brucella* CypB has a single cysteine residue in its sequence (Cys^128^) which is absent in CypA. As shown here, *Brucella* CypB has the capacity to self-associate to form homodimers. This interaction was reversed by the addition of the reducing agent DTT indicating that Cys^128^ is the amino acid responsible for dimer formation. Thus, CypB^C128M^ mutant was unable to form homodimers showing the same behavior as CypA which is only present in a monomeric state.

It has been reported that PPIases can form homo-oligomers (Zhang et al., 2011; Jakob et al., 2016; Polley et al., 2016) although, in the family of cyclophilins, only a few cases have been reported: the human hCypA (Zhang et al., 2011) and the *Trichomonas vaginalis* cyclophilin 1 (Martin et al., 2018). In addition, in the case of dimeric PPIases, no homodimer formation has been reported dependent on disulfide bonds. Hence, *Brucella* CypB is the first example of a dimeric PPIase stabilized by disulfide bridges.

Interestingly, it has been also reported that oligomerization of enzymes modulates their activities (Kropp et al., 2022). We showed here that CypB dimer formation was fully required to complement the defective phenotype of *Brucella* Δc*ypAB* mutant in stress survival, intracellular adaptation, and virulence in mice but not required for the *in vitro* PPIase activity. This apparent discrepancy can be explained because the *in vitro* PPIase substrate, the acid-denatured GFP, is not expected to be a physiological protein target. Similar results to those described here were reported for *Legionella* protein MIP, a PPIase belonging to the FKB family (Kohler et al., 2003).

As shown here, the defective phenotype of *B. abortus* Δ*cypAB* mutant cannot be complemented by CypB^C128M^ indicating that the formation of homodimers is critical for PPIase activity of CypB.

To understand in more detail how *Brucella* cyclophilins participate in the process of stress adaptation and intracellular survival, a dominant-negative experiment was performed. As shown here, the over-expression of CypB^R59A/F64A^ interfered with bacterial ability to survive to stressors and within the host cell resembling what is observed in the *B. abortus* Δ*cypAB* mutant. Interpretations of these results suggested that for the fully enzymatic activity of the dimeric CypB, both monomers must present functional active sites. As we mentioned before and it was shown previously (Roset et al., 2013), CypA and CypB are equivalent when complemented the *B. abortus* Δ*cypAB* mutant indicating a functional redundancy. Interestingly, in the dominant-negative experiment, the wild-type activity of CypA was also surprisingly inhibited, although CypA is not expected to form heterodimers with CypB^R59A/F64A^. An explanation for these results can be that homodimers formed by CypB^R59A/F64A^ or heterodimers formed by CypB-CypB^R59A/F64A^ were able to trap also the protein targets of CypA preventing their folding.

It has been reported that there is a general link between cyclophilins and cellular stress response (Dimou et al., 2017). We have described that *Brucella’s* Cyps participate in survival to diverse types of stresses which is dependent on the PPIase activity (Roset et al., 2013). Proteins involved in oxidative stress such as OxyR and Hsp33 are regulated by redox activity and activated by the formation of intramolecular disulfide bridges (Zheng et al., 1998; Graumann et al., 2001). Moreover, evidence for redox regulation of PPIase activity of cyclophilins has been reported for hCypA from T lymphocytes (Ghezzi et al., 2006), Chloroplast CypA from *Arabidopsis thaliana* (Motohashi et al., 2003), and CypA from *Schistosoma mansoni* (Gourlay et al., 2007), suggesting that redox regulation involving cysteine residues must be a common mechanism of cyclophilin regulation. In all the mentioned cases, activity regulation is mediated by the formation of intramolecular disulfide bridges. It is conceivable that in the case of *Brucella,* upon exposure to an oxidative stress condition (for instance when the bacterium enters its host cell) the intermolecular formation of disulfide bridges between two monomers of CypB can be triggered to obtain fully functional PPIase activity. Thus, the dimerization of CypB might function as a regulatory switch to respond to oxidative stress in *Brucella*.

Bacterial pathogens that have co-evolved with their host have acquired mechanisms to modulate the host cell physiology for their own benefit. Thus, pathogenic bacteria can translocate virulence proteins (known as effector proteins) into the host cell, using specialized secretion systems to hijack different processes, such as the acquisition of nutrients, vesicle trafficking, and modulation of the immune system to allow adequate time for bacterial replication. To accomplish these functions, some effector proteins use “eukaryotic-like” protein domains to mimic the structure or the function of host proteins, promoting the manipulation of a particular host cell pathway (Ke et al., 2015). In the light of, *i*) *Brucella* Cyps are overexpressed during *B. abortus* intracellular life, *ii*) *Brucella* Cyps are required for stress adaptation, intracellular survival, and virulence in BALB/c mice, *iii*) *Brucella* CypA and CypB differ in immunodominant loops that may be reflecting differential functions as well*, iv*) *Brucella* CypB has structural and functional eukaryotic characteristics, it is possible to hypothesize that CypB might function as an effector protein. Further studies to understand if CypB can function as an effector bacterial protein are still in progress.

## Conclusion

Remarkably, we have shown here that *Brucella* cyclophilins come in two different “flavors”: eukaryotic and prokaryotic. In addition, we reported here that *Brucella* cyclophilins CypA and CypB differ in various immunological and biochemical properties, despite their high degree of sequence similarity and conserved functional features. Also, we highlighted the importance of dimer formation and PPIase activity of CypB for a progressive infection in an animal model. These findings shed some light on the potential novel functions of *Brucella* Cyps, some of them could be due to the putative role of CypB as an effector bacterial protein.

## Data availability statement

The original contributions presented in the study are included in the article/supplementary material, further inquiries can be directed to the corresponding authors.

## Ethics statement

The animal study was reviewed and approved by The experimental procedure of this study (permit number CICUAE UNSAM 15/2018) was approved by the Committee on the Ethics of Animal Experiments of the Universidad Nacional de San Martín (UNSAM), under the recommendations for animal experimentation (Helsinki Declaration and its amendments, Amsterdam Protocol of welfare and animal protection and National Institutes of Health, USA NIH, guidelines: Guide for the Care and Use of Laboratory Animals).

## Author contributions

MR and GB conceived and design the experiments. EM and MR performed experiments. EM, MR, and GB analyzed the data. MR and GB wrote the paper. All authors contributed to the article and approved the submitted version.

## Funding

This work was supported by grants from the Agencia Nacional de Promoción Científica y Tecnológica, Buenos Aires, Argentina (PICT-2018-0778, PICT-2016-0412) and CONICET (PUE-0086, PIP-11220200102517CO).

## Conflict of interest

The authors declare that the research was conducted in the absence of any commercial or financial relationships that could be construed as a potential conflict of interest.

## Publisher’s note

All claims expressed in this article are solely those of the authors and do not necessarily represent those of their affiliated organizations, or those of the publisher, the editors and the reviewers. Any product that may be evaluated in this article, or claim that may be made by its manufacturer, is not guaranteed or endorsed by the publisher.

## Footnotes

^1^ https://swissmodel.expasy.org/
